# Optimizing Red Vinasse-Blue Round Scad Processing Using Integrated Dimensionality Reduction and RSM: Effects on Lipid Storage Stability

**DOI:** 10.3390/foods14183215

**Published:** 2025-09-16

**Authors:** Shan Xue, Bohu Liu, Guojin Lan, Jia Liu

**Affiliations:** 1 College of Biological Science and Technology, Minnan Normal University, Zhangzhou 363000, China; lbh30@mnnu.edu.cn (B.L.); lgj33@mnnu.edu.cn (G.L.); 2 Research Institute of Zhangzhou & Taiwan Leisure Food and Tea Beverage, Zhangzhou 363000, China; 3 Zhangzhou Food Science Research Institute, Zhangzhou 363000, China; 4 Guizhou Academy of Agricultural Sciences, Guiyang 550006, China; mcgrady456@163.com

**Keywords:** functional foods, red vinasse-blue round scads (*Decapterus maruadsi*), dimensionality reduction and RSM, processing technology, lipid stability

## Abstract

This study pioneered an intelligent process optimization framework integrating dimensionality reduction and Box–Behnken Design response surface methodology (RSM) with MATLAB R2021b(v9.11) analytics, to advance the development of functional foods from red vinasse-blue round scad. The comprehensive nutraceutical stability assessment for key functional lipids during 4 °C storage were established by systematically evaluating microwave, boiling, and foil-baking processing. The results of intelligent processing optimization showed that the optimal parameters (red vinasse addition: 2.8 g/g; processing temperature: 4 °C; processing time: 10 h) maximized the composite quality score Y (50% texture + 50% sensory), validated by MATLAB R2021b(v9.11) to achieve near-theoretical maxima. The results of functional lipid stability showed that total fat decreased significantly (*p* < 0.05), with foil-baking showing the highest loss. Partial least squares regression (PLSR) analysis revealed critical degradation of nutraceutical lipids (C20:5n-3, C22:6n-3) and an increase in saturated fats (*p* < 0.05), where boiling induced the most severe fatty acid alterations. Microwave processing accelerated lipid oxidation (highest TBARS, *p* < 0.05), compromising lipid bioactivity. The framework of red vinasse biosynthesis technology enabled precise parameter optimization, and enhanced functional component retention in underutilized fish species. This work provided a theoretical and technical foundation for intelligent manufacturing of lipid-stable nutraceuticals, positioning red vinasse—a model biosynthesis technology output—as a key ingredient for next-generation functional foods.

## 1. Introduction

As the fermentation product of monascus, the red vinasse is not only a traditional medicinal and edible food material in China, but also a famous Fujian cuisine adjuvant often eaten in the Fujian area. The red vinasse is a fermented rice product made from cooked rice (rice that has been washed and then steamed) and red yeast seed (red Aspergillus) as raw materials. It is produced through mixing and stirring, three rounds of fermentation, and three rounds of washing. The rice grains are soft and purplish red [1]. The red vinasse has a unique flavor, which can improve the storability of products [2,3].

The application of red vinasse in food has a long history, and the local residents in Fujian often use red vinasse to marinate fish and cook “red vinasse cooked meat” and “red vinasse cooked fish”. The dishes are fragrant, straightforward to eat, and not greasy. Studies have shown that red vinasse contains many functional components, such as monaskochrome, monacolin, gamma-aminobutyric acid, lovastatin, ergosterol, etc. [4,5], which have liver protection, anti-cancer, antioxidant, anti-inflammatory, anti-obesity, anti-diabetes, and other effects [6,7]. Red vinasse can play a role in meat processing, as a colorant, antibacterial, anticorrosive, and antioxidant, and has been used in many kinds of foods such as meat products, pastries, and beverages [2,3]. However, the processing of red vinasse fish is conducted mostly by empirical operation, and the study of exact technological parameters is still very lacking.

The round scads (*Decapterus maruadsi*), commonly known as balangfish or Japanese scad, are a low-value fish distributed around the world. It is the second largest marine fishing fish in our country, second only to bandfish, and an essential economic fish off the southeast coast, especially in Fujian Province [8]. It is reported that round scad tastes delicious and has high nutritional value; it is rich in polyunsaturated fatty acid (PUFA), especially eicosapentaenoic acid (EPA) and docosahexaenoic acid (DHA) [9]. However, the round scad has many disadvantages, such as soft meat, high red meat content, low pH, insufficient protein elasticity, and poor gel properties, and it deteriorates quickly after leaving the water, etc. In addition, there are limitations in traditional processing methods, most of which involve fresh fish on ice, or dried fish, and it is sold primarily as a low-value, raw or semi-processed material, and it lacks value-added processing [10,11].

Studies have shown that different processing methods have significant effects on fish quality, including processing loss, essential nutrients, fat oxidation, fatty acids composition and volatile substance composition [12,13]. Through hot processing, harmful microorganisms in fish can be reduced to ensure food safety. At the same time, the fish’s appearance, nutrition, flavor, and other edible qualities will change under high temperature conditions [14]. For instance, Yuan et al. [15] studied the influence of different cooking methods (steaming, boiling, and roasting) on the quality characteristics of golden pomfret fillet and found that the fish meat had the best color and a more diverse taste after roasting. Mi et al. [16] investigated the influence of superheated steam technology under different temperature conditions (200–300 °C) on the formation of the “crispy outside and tender inside” quality of grilled fish and the volatile flavor components. It was found that different roasting methods and temperatures have certain influences on the color and crispness of fish skin, the texture and flavor of fish meat, and other edible qualities. Among them, superheated steam has the advantage of being able to replace the traditional roasting mode in maintaining the “crispy outside and tender inside” of roasted fish. Ruan et al. [17] optimized the key processing techniques of ready-to-eat dried tangerine peel eel and analyzed the changes in storage quality of ready-to-eat dried tangerine peel eel at different storage temperatures (37 °C, 25 °C, 10 °C), thereby establishing a shelf life prediction model for the product. In addition, researchers have studied tilapia (*Oreochromis niloticus*) [12], Ctenopharyngodon idellus (*Ctenopharyngodon idellus*) [13,18], and other commonly consumed fish in China, which have been processed by different thermal methods. There have been many studies on the effects of food quality, but research on round scads cooked by the traditional processing methods has rarely been reported.

Based on this, in this paper, the red vinasse, a landmark food of Sanming City, Fujian Province, and blue round scads, a specialty of Dongshan County, Zhangzhou City, Fujian Province, were used as raw materials. Response surface combined with the MATLAB method was used to optimize the processing technological parameter of red vinasse-blue round scad. In addition, three common cooking methods (microwaving, boiling, and foil baking) were used to cook the red vinasse-blue round scad, and the influence of different processing on its lipids stability during cold storage at 4 °C were studied. The exploration of the above contents was expected not only to create an innovative impetus for the research and development of local characteristics of red vinasse-fish as a food, but also to provide a theoretical basis for the industrial production of red vinasse-blue round scad, as well as new ideas for the deep processing of low-value fish. Moreover, this research can provide an innovative, intelligent optimization strategy for the development of marine functional foods rich in high-quality oils and with excellent storability, organically integrating targeted processing with nutritional health and storage.

## 2. Materials and Methods

### 2.1. Preparation of Blue Round Scad Processed with Red Vinasse

**Figure 1:**
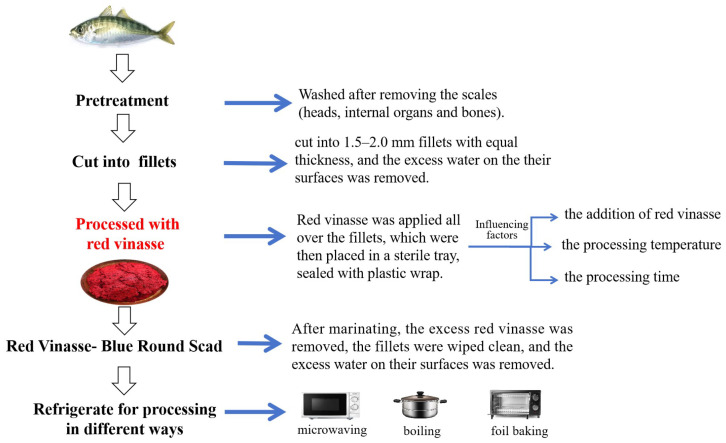
The specific operation flow of preparation of red vinasse-blue round scad.

Blue round scads (purchased from RT-Mart, Zhangzhou, China) processed with red vinasse (produced in Youxi County, Sanming City, China), had a water content of 74.01 ± 0.26%, the content of total sugar was 3.92 ± 0.25%, the content of crude fat was 2.21 ± 0.16%, the protein content was 10.8 ± 0.22%, the total phenol content was 365.43 ± 1.37 μg/g, and the IC_50_ value was 20.16 mg/mL). They were prepared using a method commonly employed by residents to marinate fish. The specific operation flow is shown in Figure 1.

### 2.2. Single Factor Experiment on Processing Technological Parameter of Red Vinasse-Blue Round Scad

The effects of different quantities of red vinasse added (0.6, 1.2, 1.8, 2.4, and 3.0 g/g based on the weight of the fish meat), different processing temperatures (0, 4, 8, 12, and 16 °C), and processing times (3, 6, 9, 12, and 15 h) on the texture, composite score, and sensory score of blue round scads were investigated. All the chemical reagents were purchased from Xilong Science Co., Ltd., Shantou City, China.

### 2.3. Determination of Texture Composite Score and Sensory Scores

#### 2.3.1. Determination of Texture Composite Score (Y1)

The hardness, cohesion, elasticity, and chewability of the samples were determined by a textural instrument (CT3-10K, Brookfield Corporation, Middleboro, MA, USA) [19]. The test type was TPA texture analysis, the test target type was distance, the target value was 2.0 mm, the waiting time was 0 s, the trigger point load was 1.00 N, and the test speed was 1.00 mm/s. The probe model was TA18, and the cycle number was 2. The composite score of texture (hardness, cohesion, elasticity, and chewability) of the samples was analyzed by a dimensionality reduction method based on the Kaiser–Meyer–Olkin (KMO) and Bartlett sphericity test [20].

#### 2.3.2. Determination of Sensory Scores (Y2)

The sensory evaluation of red vinasse-blue round scad was carried out according to the scoring criteria in Table 1. Sensory evaluation was conducted with 10 trained panelists, with a 1:1 ratio of males to females, from the College of Biological Science and Technology, Minnan Normal University [21]. The different products were numbered randomly and distributed to the 10 panelists. The panelists assessed the color, fragrance, and tissue of the samples according to the appearance, color, aroma, unpleasant odor, and tissue.

### 2.4. The Response Surface Design Combined with MATLAB Analysis

According to the results of single-factor experiments, the three-dimensional interaction of factors was analyzed with the combined texture composite score (Y1) and sensory score (Y2) as the double response values, and the total response value (total composite score) (Y) was expressed as the average value of Y1 and Y2. At the same time, MATLAB software R2021b(v9.11) was used to optimize the four-dimensional interactive effects of the addition of red vinasse (A), processing temperature (B), and processing time (C) on texture composite score (Y1), sensory score (Y2), and total composite score (Y) (50% Y1 + 50% Y2) of red vinasse-blue round scad through code programming and graphic processing. The factors and levels of the Box–Behnken response surface design combined with MATLAB test are shown in Table 2.

### 2.5. The Influence of Different Processing on the Lipids Stability of Red Vinasse-Blue Round Scad During Storage

#### 2.5.1. The Different Processing Methods

The fillets of the red vinasse-blue round scad were processed using three common home cooking methods. Method 1: the microwaving processing conditions were medium-high fire for 1 min. Method 2: the foil baking conditions used the upper and lower fire at 160 °C. Method 3: the baking time was 5 min, and the boiling conditions were a boiling water bath for 90 s (the center temperature of the fish fillet was about 80 ± 3 °C). The unprocessed red vinasse-blue round scad was used as the control group.

#### 2.5.2. Extraction and Determination of Total Fat

The total fat extraction procedure referred to the method in Xue et al. [22]. Ten grams of blue round scad was minced and placed in a conical bottle, and 120 mL of chloroform methanol solution with a volume ratio of 2:1 (*v*/*v*) was added. After fully shaking, the sample was bathed in a constant-temperature water bath at 45 °C for 2.5 h, then filtered. After filtration was completed, 30 mL of saturated NaCl solution was added to the filtrate, and the liquid was fully shaken and transferred to a liquid separation funnel for static stratification. The lower layer of fat extract was collected in the separator funnel, filtered and dried with anhydrous Na_2_SO_4_, and concentrated in a 45 °C water bath with a rotary evaporator. Finally, the crude fat sample was obtained. The total fat content was calculated according to Formula (1):

(1) $$\mathrm{Content}\;\mathrm{of}\;\mathrm{total}\;\mathrm{fat}\;\left(\%\right)=\frac{m_{2}-m_{1}}{m}\times 100$$

where m_2_: the mass of total fat and conical bottle (g); m_1_: the mass of conical bottle (g); and m: the sample weight (wet weight) (g).

#### 2.5.3. Determination of Thiobarbituric Acid Reactive Substances (TBARS)

The lipid oxidation at 4 °C stored for 0 h, 24 h, 48 h, 72 h, 96 h, and 120 h of red vinasse-blue round scad processed by the above three different methods was investigated. The determination of TBARS referred to the method of Xue et al. [19]. Samples of blue round scad processed with red vinasse were weighed, crushed, and placed in a conical flask (Company: Chengdu Shuniu Glassware Co., Ltd., Origin: Chengdu, China, Specification: Ground glass, Volume: 250 mL) with 50 mL of distilled water and 50 mL of 10% trichloroacetic acid solution, then filtered at 27 °C after constant-temperature shaking for 1 h, and the filtrate was used. To 8 mL of the filtrate supernatant, 2 mL 0.06 mol/L of thiobarbituric acid (TBA) solution was added. The mixture was shaken well and placed in a constant-temperature water bath at 80 °C for 2 h, then removed and cooled for 30 min to room temperature. Colorimetric measurements were performed at 532 nm, and the absorbance was recorded [21]. Each group of samples was measured in parallel 3 times, and the average value was selected (as shown in Formula (2)).

(2) $$\mathrm{TBARS}\;\left(\mathrm{mg}\frac{\mathrm{MDA}}{\mathrm{kg}}\right)=\frac{c\times V\times 1000}{m\times 1000}$$

where *c* represents the concentration of malondialdehyde in the sample solution obtained from the standard series curves/(μg/mL); *V* stands for constant volume of sample solution/mL; and m represents the sample mass (g).

#### 2.5.4. Determination of Fatty Acid Composition

The composition of fatty acids was determined by a 7890 B gas chromatographic instrument (produced by Agilent Technologies, Inc., Stevens Creek Boulevard, Santa Clara, CA 95051, USA) with flame ionization detector and SH-RtTM-2560 Column (Column 100 m, 0.25 mm ID, 0.20 μm film thickness, produced by Shimadzu Corporation, Nishinokyo-Kuwabaracho, Nakagyo-ku, Kyoto 604-8511, Japan) with a shutter ratio of 10:1 (*v*/*v*). The sample size was 1 µL, and the carrier gas was nitrogen. The flow rate was 20 mL/min, the inlet temperature was 250 °C, and the detector temperature was 260 °C. The heating procedure for the column box was as follows: the initial temperature was 140 °C, kept for 1 min, then increased to 240 °C at 4 °C/min and maintained for 19 min. The retention time of 37 mixed fatty acid methyl ester standards was compared for qualitative analysis of fatty acids, and area normalization was used for quantitative analysis. The polyunsaturated fatty acid (PUFA), saturated fatty acid (SFA), and monounsaturated fatty acid (MUFA) content was expressed as the relative proportion (%) of individual fatty acids to total fatty acids [22].

### 2.6. Statistical Analysis

The experiment was repeated three times, and the results were expressed as mean ± standard variance. ORIGIN 8.5 (OriginLab Corp., Northampton, MA, USA) was used for mapping, and SPSS Statistics 24.0 (IBM^®^ SPSS Statistics, Armonk, New York, NY, USA) was used to test the significant differences between the results (*p* < 0.05) and multiple comparisons. Design-Expert 8.0.6 Trial software was used for response surface optimization experiments, and MATLAB software R2021b(v9.11) was used for interactive test data calculation and four-dimensional plotting. Excel software was used for analysis and drawing of data correlation. The PLSR was analyzed using Unscrambler 9.7 software (CAMO ASA, Trondheim, Norway). All data were standardized using SPSS 24.0 software before analysis.

## 3. Results and Discussion

### 3.1. Establishment of Evaluation Model of Texture Composite Score of Red Vinasse-Blue Round Scad

#### 3.1.1. The Suitability Test

**Figure 2:**
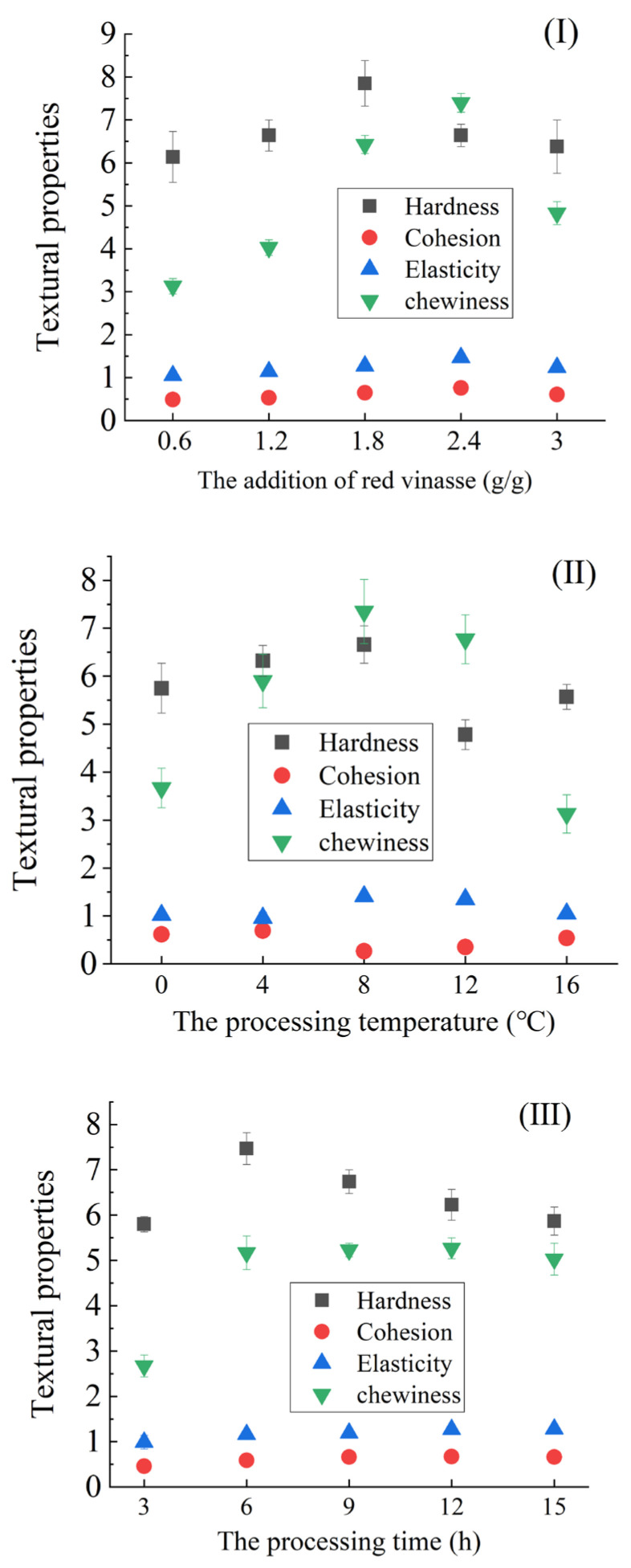
The influence of different factors on texture properties of red vinasse-blue round scad ((**I**): the influence of addition of red vinasse; (**II**): the influence of processing temperature; and (**III**): the influence of processing time).

According to the experimental design in Section 3.1.1, the influence of different factors on the texture composite score of red vinasse-blue round scad were shown in Figure 2. As shown in Figure 2, the optimal overall performance was observed at a red vinasse addition ratio of 1:1.8 (g/g), where hardness and chewiness reached, significantly, the highest values (*p* < 0.05). At the same time, cohesion and elasticity also demonstrated superior levels. Regarding processing temperature, both hardness and chewiness were notably enhanced at 8 °C, though cohesion decreased significantly. Chewiness dropped to the minimum at 16 °C. When processing time was extended to 6 h, hardness peaked, and cohesion, elasticity, and chewiness all improved significantly and remained stable from 6 to 15 h (*p* < 0.05). In conclusion, the synergistic combination of red vinasse addition at 1:1.8 (g/g), 8 °C processing temperature, and ≥6 h processing duration collectively optimized the textural properties of the product.

Generally, when the KMO statistic was greater than 0.5, it was considered that factor analysis could be performed [23,24]. The KMO and Bartlett spherality tests of the dimensionality reduction models of hardness, cohesion, elasticity, and chewiness of the texture indexes of the samples are shown in Table 3. It can be seen from the results that the KMO value of the factor test was 0.596, which was greater than the standard 0.5 given by statistical Kaiser, so the evaluation model of the texture composite score of red vinasse-blue round scad was suitable for factor analysis.

#### 3.1.2. Total Variance of Interpretation

**Figure 3:**
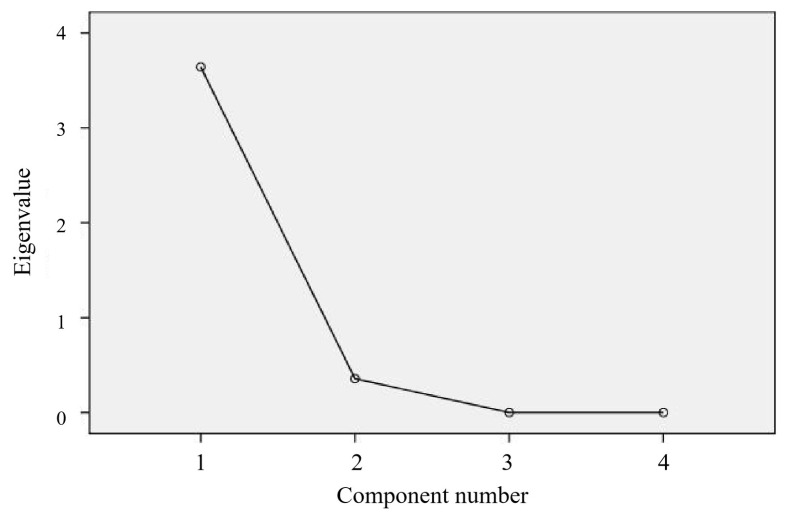
The scree plot of factor analysis.

The SPSS 24.0 software was used for data processing, and principal component analysis was used to extract factors with an eigenvalue greater than 1. The output result of the scree plot of factor analysis was shown in Figure 3. It can be seen from the figure that only one factor of the eigenvalue curve was greater than 1. After variance maximization orthogonal rotation, the cumulative variance contribution rate of one factor reached 91.577%, which meaned, and the extracted factor reflected the information of 91.577% of the original four indicators, as shown in Table 4.

#### 3.1.3. Extraction of Common Factors to Establish Comprehensive Index Calculation

By factor rotation, each variable only had a significant load on one common factor, while the load on the other common factors was relatively small. By highlighting the relationship between each common factor and those variables with a large load, the common factor can be reasonably explained through these variables with a large load. The formula for calculating the total composite score (Y) according to the rotating load matrix was as follows:

Y = 0.266 X_1_ + 0.244X_2_ + 0.262X_3_ + 0.271X_4_

where X_1_ represented hardness, X_2_ represented cohesion, X_3_ represented elasticity, and X_4_ represented chewability.

### 3.2. The Influence of Different Factors on Texture Composite Score of Red Vinasse-Blue Round Scad

#### 3.2.1. The Influence of the Addition of Red Vinasse on Texture Composite Score

**Figure 4:**
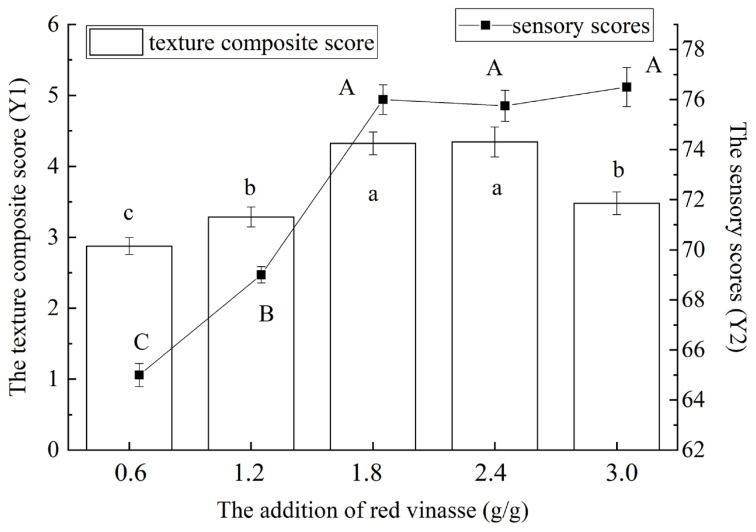
The effect of the red vinasse addition on the texture composite score (Y1) and sensory score (Y2) of blue round scad. (a–c: different lowercase letters represented significant differences between the data of texture composite score (*p* < 0.05); A–C: different capital letters data represented significant differences between the data of the sensory score (*p* < 0.05)).

The effect of addition of red vinasse on the texture composite score (Y1) and sensory score (Y2) of blue round scad are shown in Figure 4. When the supplemental level of red vinasse was 1.8 g/g, the samples had the maximum texture composite score, and there was no significant difference between the samples of supplemental level of 1.8 g/g and 2.4 g/g (*p* > 0.05). When the supplemental level of red vinasse was 3.0 g/g, the sensory scores of red vinasse-blue round scad were the highest, but there was no significant difference between the samples of supplemental levels of 1.8 g/g, 2.4 g/g and 3.0 g/g (*p* > 0.05). By comprehensive consideration, the addition of red vinasse was selected to be 2.4 g/g. This may be because red grains contain many active antioxidant ingredients, which can inhibit the deterioration of fish to a certain extent, so as to maintain good properties of texture (hardness, cohesion, elasticity, and chewability) and sensory properties [6,25].

#### 3.2.2. The Influence of Processing Temperature on Texture Composite Score

**Figure 5:**
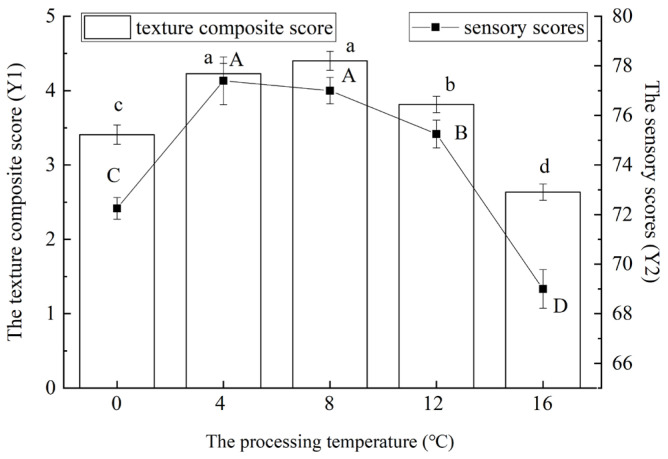
The effect of processing temperature of red vinasse on the texture composite score (Y1) and sensory score (Y2) of blue round scad. (a–d: different lowercase letters represented significant differences between the data of the texture composite score (*p* < 0.05); A–D: different capital letters data represented significant differences between the data of the sensory score (*p* < 0.05)).

As shown in Figure 5, the processing temperature significantly influenced the hardness, cohesiveness, springiness, and chewiness of red vinasse-blue round scad (*p* < 0.05). Figure 5 indicated that both the comprehensive texture profile score and sensory evaluation score of the samples initially increased and subsequently decreased with rising processing temperatures (*p* < 0.05). This pattern may be attributed to enhanced Monascus purpureus fermentation activity at higher marination temperatures. The intensified fermentation caused fluctuations in the fish’s hardness, springiness, and chewiness while accelerating protein degradation, thereby amplifying the impact on textural properties [26]. The highest comprehensive texture score was observed at 8 °C, whereas the peak sensory score occurred at 4 °C—though this value showed no significant difference from the score at 8 °C (*p* < 0.05). Overall, samples marinated within the 4–12 °C range exhibited substantially higher texture and sensory scores. Considering these results collectively, 8 °C was selected for subsequent experiments.

#### 3.2.3. The Influence of Processing Time on Texture Composite Score

**Figure 6:**
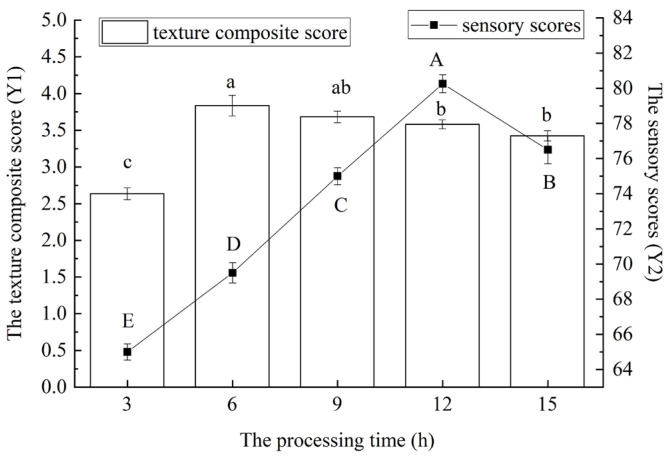
The effect of processing time of red vinasse on the texture composite score (Y1) and sensory score (Y2) of blue round scad. (a–c: different lowercase letters represented significant differences between the data of the texture composite score (*p* < 0.05); A–E: different capital letters data represented significant differences between the data of the sensory score (*p* < 0.05)).

As can be seen from Figure 6, with the extension of processing time, the texture composite score of red vinasse-blue round scad first showed an overall increase, and then decreased (*p* < 0.05). When the processing temperature was 6 h, the texture composite score of samples reached the highest, which had no significant difference from the results at 9 h (*p* > 0.05). This may be because the cohesiveness, elasticity, and chewability of the sample also increased with the extension of the processing time, and the hardness first increased and then decreased (Figure 2). The effect of the processing time on sensory scores was also significant (*p* < 0.05), and the maximum value was found at 12 h. By comprehensive consideration, the processing time of 12 h was selected. On this condition, the overall score of texture was higher and the best sensory score was obtained [27].

### 3.3. Results of Box–Behnken Design Combined with MATLAB Analysis

#### 3.3.1. Establishment of Response Surface Model of Box–Behnken Design

On the basis of single-factor experiment and according to the experimental principle of Box–Behnken Design, the texture composite score (Y1) and sensory score (Y2) were determined. The Y value (50% Y1 + 50% Y2) was used as the index, and the addition of red vinasse (A), the processing temperature (B), and the processing time (C) were selected as the investigation factors. According to the Design test of Design-Expert 8.0 software, the values of each factor were shown in Table 2, and the optimization results are shown in Table 5. By analyzing the data in the table, the regression equation model of Y was obtained as follows:

Y = 37.82 − 0.56A − 2.75B − 2.52C − 0.54AB − 2.34AC + 0.15BC + 2.40A^2^ + 0.91B^2^ + 1.23C^2^ (3)

#### 3.3.2. Significance Test of Response Surface Model of Box–Behnken Design

The variance analysis was performed on the regression model and a significance test was conducted on the model coefficients. According to the results shown in Table 6, the regression equation model was extremely significant (*p* < 0.01), indicating that the independent variable and dependent variable in the regression equation had extremely significant correlation. Among them, the primary terms B and C, interaction terms AC, and quadratic terms A^2^ in the regression equation were extremely significant (*p* < 0.01), and the above variables had significant effects on Y. The non-significant missing item (*p* > 0.05) indicated that the proportion of non-normal errors in the equation and the actual fitting was small, and the equation indicated a good regression equation relationship between the dependent variable and the independent variable. Among the correlation coefficient R^2^ = 0.9379, regression equation correction coefficient R^2^_Adj_ = 0.8581, signal-to-dryness ratio AP = 11.065, and coefficient of variation = 3.21%, the closer the R^2^ value was to 1, the more reliable the regression equation was, indicating that the regression equation can truly reflect the relationship between the factors of the experiment and the response value. The R_Adj_^2^ value showed that 85.81% of the response value can be explained by the regression equation after correction. AP was greater than 4, indicating that the equation had a good fit and reliability. The C.V. value reflects the confidence of the regression equation, and the smaller the value, the higher the confidence of the regression equation. Therefore, the regression equation had high confidence [28,29].

#### 3.3.3. Analysis of the Result of Box–Behnken Design

**Figure 7:**
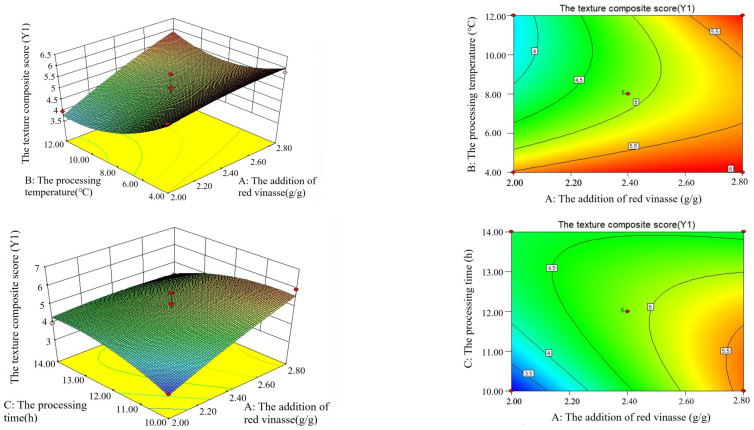

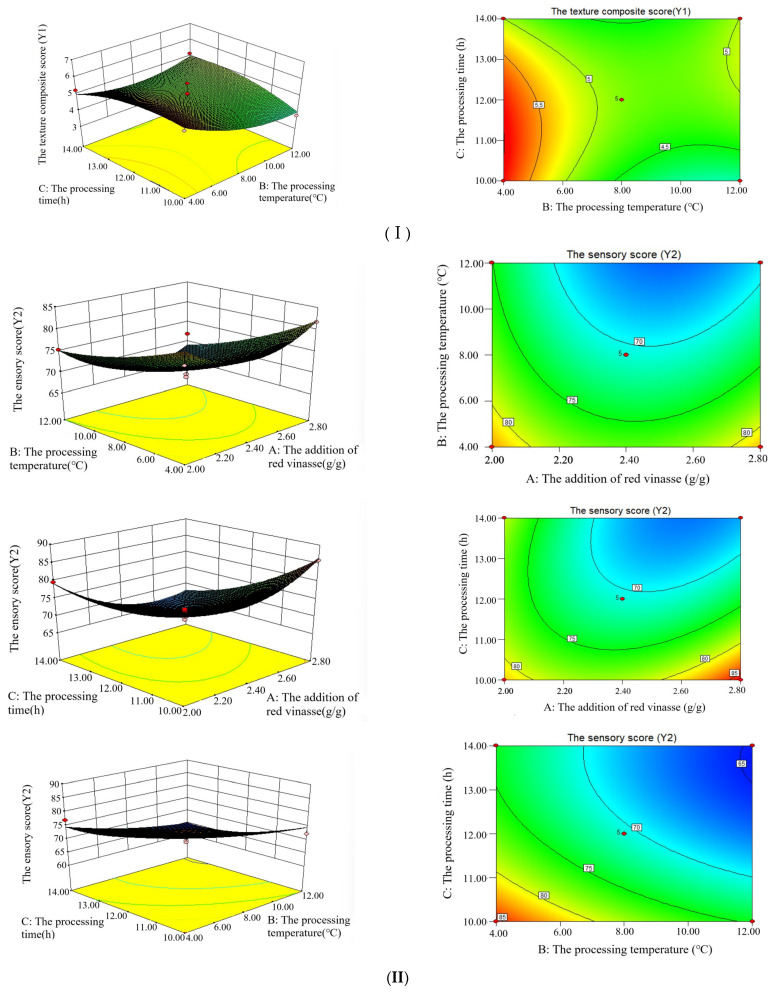

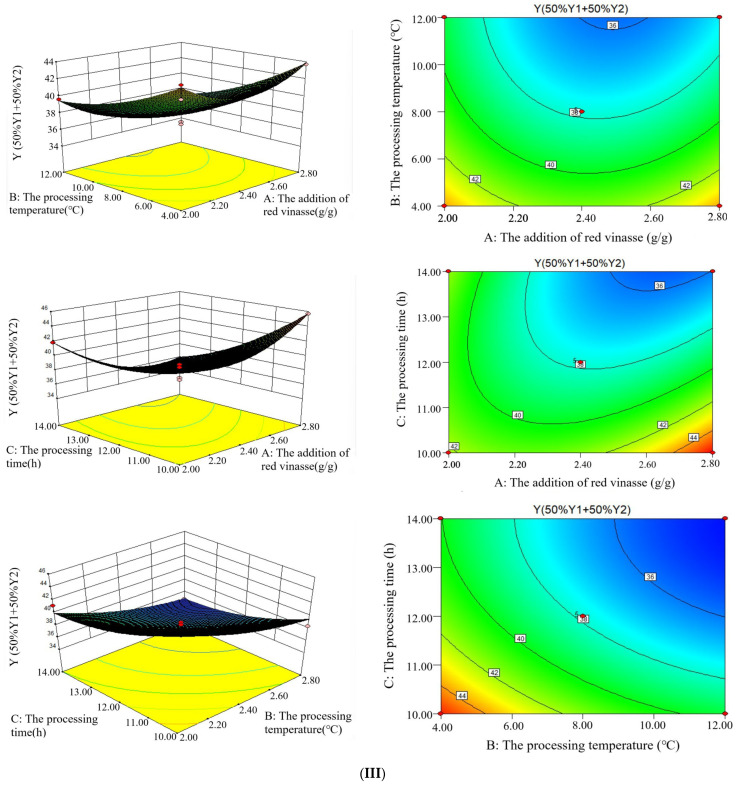
Contour lines and response surface diagrams of the interaction of various factors. (**I**) Contour lines and response surface plots of the interaction of various factors based on Y1. (**II**) Contour lines and response surface plots of the interaction of various factors based on Y2. (**III**) Contour lines and response surface plots of the interaction of each factor based on Y (50% Y1 + 50% Y2). The numbers on the contour lines represent the magnitude of the response value (Y value). The warmer the color (such as red or yellow), the higher the response value is usually. The colder the color (such as blue or purple), the lower the response value it usually indicates.

Through the Box–Behnken design, based on Y1, Y2, and Y (50% Y1 + 50% Y2) of red vinasse-blue round scad, the interaction analyses of the addition of red vinasse (A), processing temperature (B), and processing time (C) were conducted respectively. The results are shown in Figure 7.

As shown in Figure 7I, the contour lines and response surfaces of the interactions among various factors indicated that the interaction effect between A (the addition of red vinasse) and B (processing temperature) on Y1 was the most significant (the contour lines were clearly elliptical), while the interaction between A and C (processing time) followed. As can be seen from Figure 7II, the interaction between B and C had a significant impact on Y2. The Y2 value was higher when combined with low temperature (4–10 °C) and short time (8–12 h). It can be seen from Figure 7III that the interaction between A and B dominated the change of the Y value. When A increased to 2.8 g/g and B was 4 °C, the Y value showed a peak trend.

Based on the influence of each factor on Y1 and Y2, the impact of the interaction of two factors, namely the addition of red vinasse (A), the processing temperature (B), and processing time (C), on the comprehensive score Y (50%Y1 + 50%Y2) was analyzed emphatically (Figure 7III). As can be seen from the figure, the response surface graph of the interaction between the addition of red vinasse (A) and the processing time (C) had a relatively steep slope, and the contour graph was elliptical. This indicated that the interaction between A and C had a significant impact on the comprehensive score (Y) of red vinasse-blue round scad (*p* < 0.05). When the processing time (C) was fixed, with the increase of the addition of red vinasse (A), the Y value showed a gradually increasing trend. With A fixed addition of red vinasse (A), as the curing time (C) increased, the Y value showed an overall downward trend. However, the response surfaces of the interaction between the addition of red vinasse (A) and the processing temperature (B), as well as the interaction between the processing temperature (B) and the processing time (C), were relatively gentle, indicating that the interaction between A and B, and between B and C was not significant (*p* > 0.05).

#### 3.3.4. Matlab Analysis of Four-Dimensional Interaction

**Figure 8:**
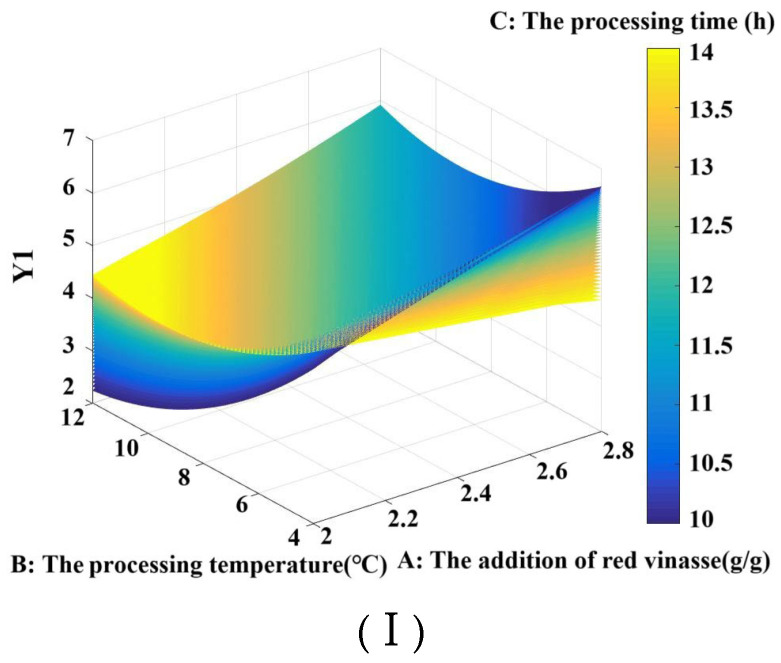

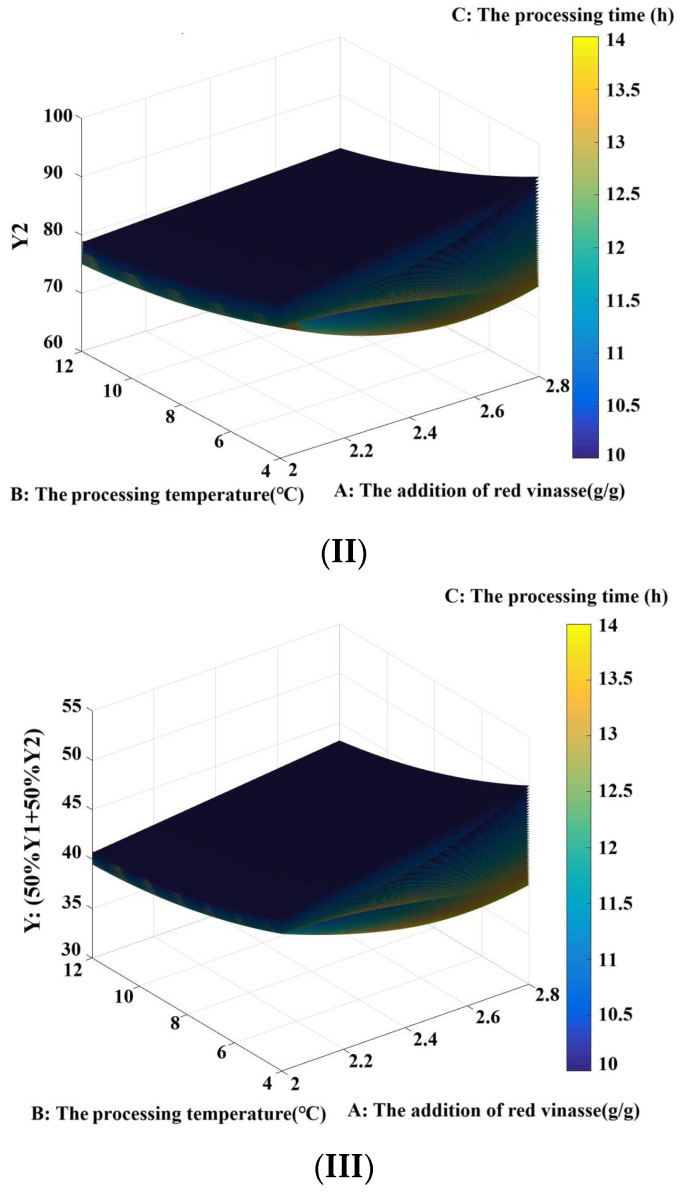
The 4-D interactive surface based on the optimization of Y1 value (**Ⅰ**), Y2 value (**II**), and Y(50% Y1 + 50% Y2) (**III**).

Through programming, four-dimensional effect diagrams illustrating the interactive effects of red vinasse addition (A), processing temperature (B), and processing time (C) on sample Y1, Y2, and Y (where Y = 50% Y1 + 50% Y2) were generated and are shown in Figure 8I–III, respectively. Program analysis determined the theoretical maximum values: Y1 max = 6.6583, Y2 max = 94.2695, and Y max = 50.1000. Matrix computation yielded the optimal processing parameters for red vinasse-blue round scad as follows: red vinasse addition of 2.8 g/g, processing temperature of 4 °C, and processing time of 10 h.

**Figure 9:**
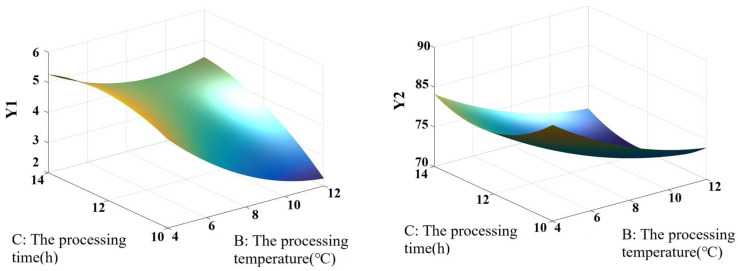

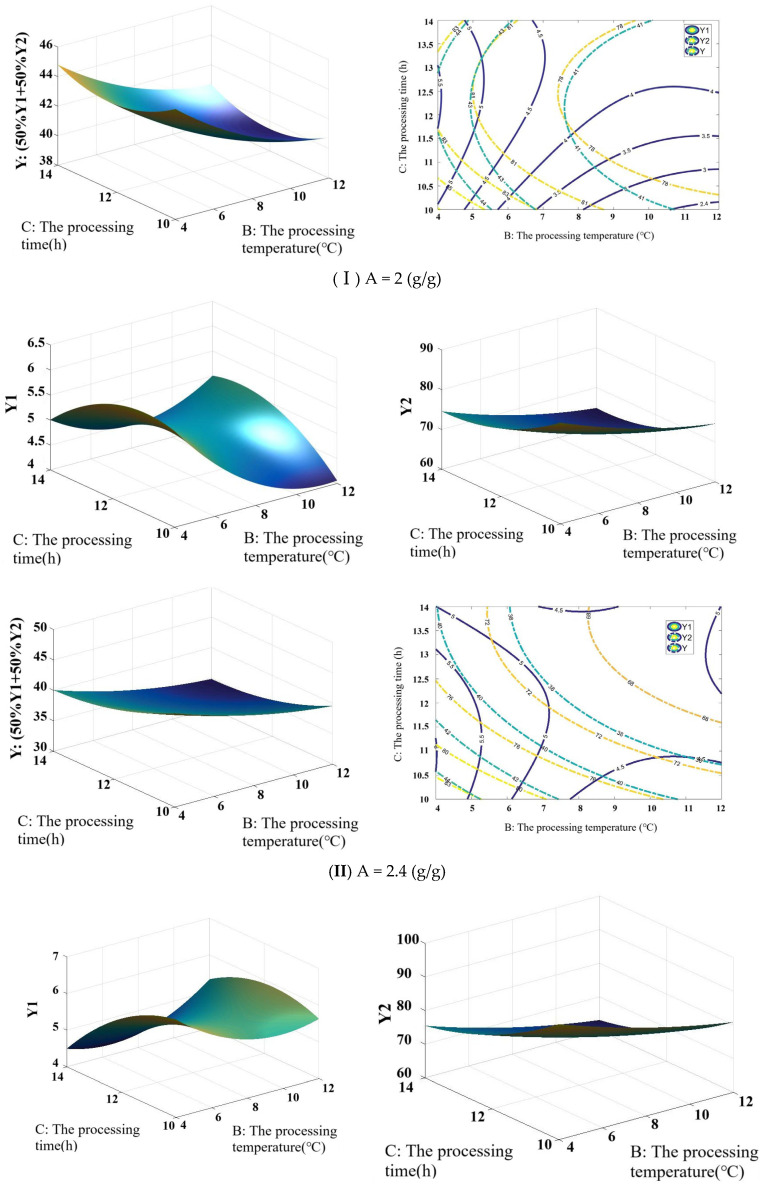

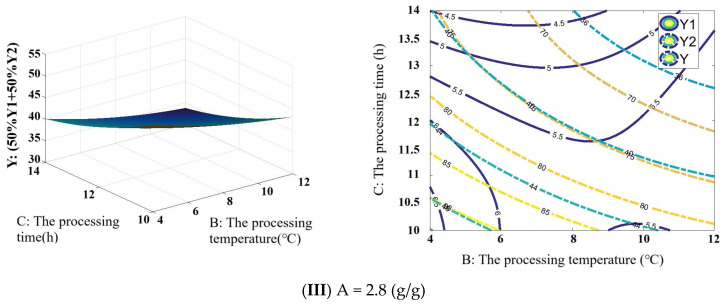
The 3-D interactive surface based on the optimization of Y1 value, Y2, and Y(50% Y1 + 50% Y2).

To better describe and analyze the interactive effects between the data, three-dimensional rotating surface plots and contour projection plots depicting the interactive effects of processing temperature and time on Y1, Y2, and Y were drawn for low (2 g/g), medium (2.4 g/g), and high (2.8 g/g) red vinasse addition levels (Figure 9).

When the red vinasse addition (A) was set at low level (2 g/g): with processing temperature (B) fixed, Y1 showed no regular change as processing time (C) increased. With processing time (C) fixed, Y2 gradually decreased as temperature (B) increased. The change trend of Y was consistent with that of Y2 (Figure 9Ⅰ). At this point, the value ranges were as follows: Y1 ranged from 2.432 to 5.5480, Y2 ranged from 75.0362 to 87.1145, and Y3 ranged from 39.4762 to 45.4725. Y1 and Y2 could simultaneously achieve relatively high values when the processing temperature was between 4~6 °C and the processing time was between 10~11 h.

When the red vinasse addition (A) was set at the medium level (2.4 g/g), the changing trends of Y1 and Y2 were consistent with those observed under the same addition level. At this setting: Y1 ranged from 4.0453 to 6.0131, Y2 ranged from 64.7799 to 85.4525, and Y ranged from 34.8466 to 45.3912. Y1 and Y2 were able to simultaneously achieve relatively high values when the processing temperature was between 4–5 °C and the processing time was between 10–10.5 h (Figure 9II).

When red vinasse addition (A) was set to its upper limit (2.8 g/g), with processing temperature (B) fixed, Y1, Y2, and Y exhibited an overall downward trend as processing time (C) increased. Under these conditions: Y1 ranged from 4.2846 to 6.6583, Y2 ranged from 63.1245 to 94.2695, and Y ranged from 33.8025 to 50.1000. Y1 and Y2 could simultaneously achieve relatively high values when the processing temperature was between 4–6 °C and processing time was within 10–11 h (Figure 9III).

In summary, when red vinasse addition was at its upper limit (A = 2.8 g/g), Y1, Y2, and Y were all able to achieve their theoretical higher values. The values of Y1, Y2, and Y approached their maxima as the processing temperature approached 4 °C and the processing time approached 10 h. This finding is consistent with the conclusions from the response surface analysis. The process of using red vinasse in fish processing was a complex biochemical reaction. The nutritional composition of red vinasse, residual dominant microorganisms, and their metabolites may affect the physicochemical properties of fish, consequently altering its textural quality and sensory characteristics [30,31,32,33]. Within this complex system, identifying the primary biochemical factors involved remains a topic for future in-depth investigation.

#### 3.3.5. Verification Test

It was verified that under the conditions of 2.8 g/g addition of red vinasse, processing temperature at 4 °C and processing time for 10 h, the texture composite score (Y1) was 6.25 ± 0.31, sensory score (Y2) was 92.43 ± 0.82, and total composite score (Y) was 49.34 ± 0.56. There was no significant difference between the model and the theoretically predicted value (50.10) (*p* > 0.05), indicating that the model was reasonable and reliable. Furthermore, the MATLAB analysis confirmed that when red vinasse addition reached its upper limit (A = 1:2.8 g/g), Y1, Y2, and Y could all achieve their theoretical higher values. As the processing temperature approached 4 °C and the processing time approached 10 h, the values of Y1, Y2, and Y values moved closer to their maxima, which was consistent with optimization conclusions. The integrated optimization approach combining the Box–Behnken Design response surface methodology with MATLAB not only accurately determined the optimal extraction protocol but also visually identified reasonable processing parameter ranges. This provided both a theoretical foundation and innovative insights for red vinasse-blue round scad processing and industrial utilization research.

### 3.4. Effects of Different Processing Methods on Total Fat Content of Red Vinasse-Blue Round Scad

**Figure 10:**
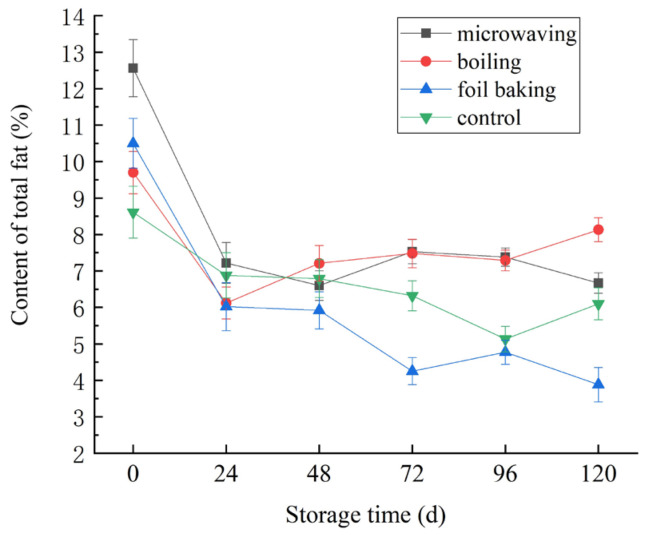
Effects of storage temperature on total fat content of red vinasse-blue round scad processed in different ways.

As can be seen from Figure 10, during the refrigeration period at 4 °C, the total fat content of red vinasse-blue round scad by different processing showed an overall trend of decline (*p* < 0.05). Among them, the change of total fat content in the control group (non-processing) was relatively uniform and gentle, while the crude total fat content of the samples by microwaving, boiling, and foil baking decreased sharply at the initial stage of storage, and the decline trend tended to be gentle at the later stage. The effect of foil baking on the total fat content of samples was more obvious (*p* < 0.05). It was speculated that the reason for this phenomenon was that hot processing will aggravate the oxidation and decomposition of total fat to a certain extent, and with the extension of storage time, the samples will undergo oxidation and decomposition under the action of microorganisms and their enzymes [32]. In addition, the oxidation of fat has previously been shown to relate to the water content, and the cooking loss after different processing and the drip loss during storage may cause changes in the water content of the sample, and then the proportion of total fat also changed accordingly [10,33].

### 3.5. Effects of Different Processing Methods on TBARS of Red Vinasse-Blue Round Scad

**Figure 11:**
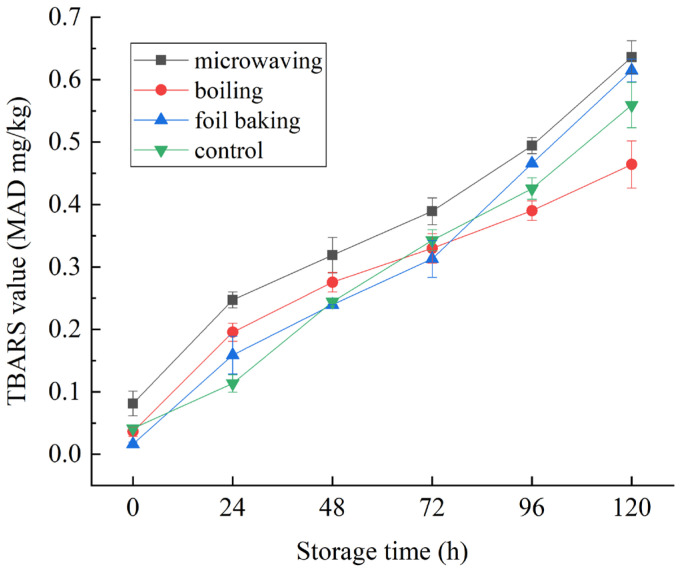
Effects of storage time on TBARS of red vinasse-blue round scad processed in different ways.

Thiobarbituric acid reactant value (TBARS) is a common standard for evaluating the quality of meat products during storage, and is often expressed by the content of malondialdehyde (MDA) in samples per kilogram [34]. As can be seen from Figure 11, the TBARS values of samples in all groups were significantly increased with the extension of storage time (*p* < 0.05). Among them, the MDA production in microwave treatment group was the highest and the fastest. TBARS in the cooking group increased rapidly in the early stage and were more uniform in the later stage. The MDA production in the tin foil baking group increased sharply from 72 to 120 days. In the control group, the increase of TBARS was relatively stable. According to the recommended limit standard of TBARS value 0.5 mg MDA/kg in the literature [35,36], Caranx was basically in line with the standard within 96 h after different thermal treatments. On the whole, in terms of lipid oxidation, the storage capacity of the experimental group and the control group was better. It was speculated that this was related to the functional components in red vinasse, such as red yeast rice pigment and lovastatin, which had antibacterial, antiseptic, and antioxidant functions [37,38].

### 3.6. Effects of Different Processing Methods on Fatty Acid Composition of Red Vinasse-Blue Round Scad

The changes of fatty acids composition of red vinasse-blue round scad processed by microwaving, steaming, foil baking, and untreated are shown in Table 7. The content of fatty acid was expressed as the relative proportion (%) of individual fatty acids to total fatty acids. By GC analysis, 22 kinds of fatty acids were identified, including 6 kinds of SFA, 5 kinds of MUFA, and 11 kinds of PUFA. Compared with the control group (non-processing), the percentages of SFA and MUFA significantly increased after different processing (*p* < 0.05); in particular, the percentages of C16:0 and C18:1n-9 significantly increased after tin foil baking and microwave processing (*p* < 0.05). The proportion of PUFA in all experimental groups had a significant decreasing trend, and the decrease rate in the microwave group was greater than that in the other two groups (*p* < 0.05), especially the components of C18:2n-6 and C22:6n-3.

**Figure 12:**
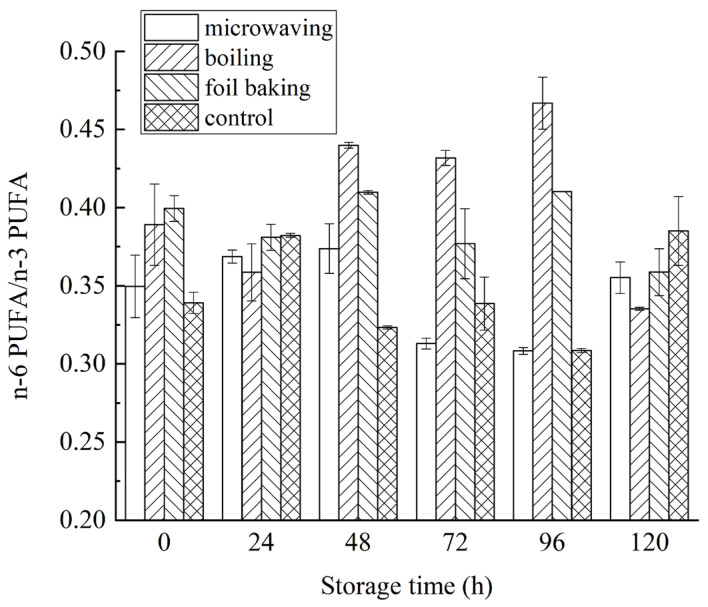
Effects of storage time on n-6/n-3 PUFA ratio of red vinasse-blue round scad processed in different ways.

In order to further analyze the effects of different processing methods on the nutritional composition of PUFA, the changes of n-6 PUFA/n-3 PUFA ratio of samples from each group during storage are shown in Figure 12. As can be seen, the ratio of n-6 PUFA/n-3 PUFA of fatty acids in all groups was low, and the ratio of n-6 PUFA to n-3 PUFA of fatty acids after microwaving, boiling, and foil baking did not show significant regular changes during 120 h at 4 °C refrigeration. As for the three standard treatments, the n-6 PUFA/n-3 PUFA ratio ranged from 0.31 to 0.39 in the microwave group, 0.34 to 0.39 in the cooking group, 0.36 to 0.40 in the foil baking group, and 0.34 to 0.38 in the control group. During the storage period, the n-6 PUFA/n-3 PUFA ratio of all groups showed an increasing trend with the extension of time (*p* < 0.05). It had been reported that when the dietary n-6 PUFA/n-3 PUFA ratio was less than 4, it can significantly reduce the risk of suffering from “chronic diseases of life”, such as coronary disease and cancer [39,40]. To a certain extent, the lower the ratio of n-6 PUFA/n-3 PUFA diet, the more obvious the resistance to many chronic diseases, and the types of diseases targeted by different ratios vary [41,42]. Therefore, it can be seen that the PUFA nutrient composition of red vinasse-blue round scad in different treatments was good and recommendable.

It was evident that the red vinasse-blue round scad possessed a favorable fatty acid profile, being rich in n-3 PUFAs such as EPA and DHA, which can effectively optimize the dietary fatty acid intake ratio in modern diets. Its potential health benefits include assisting in reducing the risk of cardiovascular diseases, promoting neural and visual development in infants and young children, and exerting positive effects such as anti-inflammatory and cognitive-enhancing properties [40,41]. This work significantly enhanced the value of underutilized fish species, providing a scientific basis and practical model for developing marine-derived functional foods targeting specific health needs (e.g., heart and brain health). It holds substantial practical significance for advancing the transformation of a nutrition-oriented agricultural food system and improving consumer health.

### 3.7. PLSR Analysis of Fatty Acid Composition of Red Vinasse-Blue Round Scad by Different Processing

**Figure 13:**
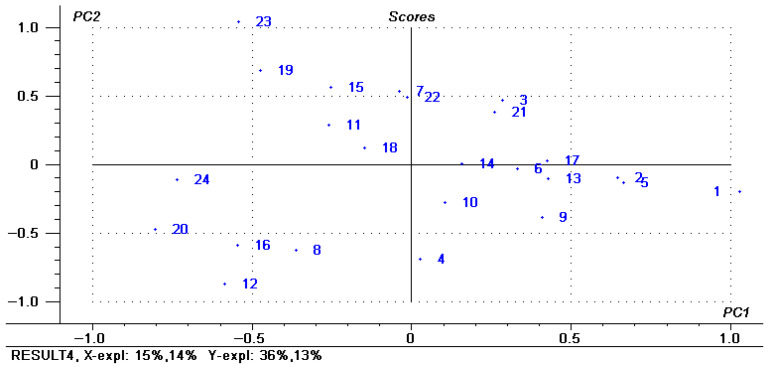
Score chart of correlation of PLSR indicators.

PLSR was a relatively new multivariate statistical data analysis method, which combined the advantages of principal component analysis, canonical correlation analysis, and multiple linear regression analysis [43]. At present, the PLSR has been applied to the analysis of changes in fatty acid composition of rabbit meat [22], changes in the rule of fatty acid deposition in rabbit meat [44], the distinction between low-fat and full-fat yogurt [45], and the detection of pork sausage [46]. In order to more clearly describe the effects of different processing methods and storage time on the changes of composition of fatty acids of red vinasse-blue round scad, the PLSR was used to establish a mathematical model. It can be seen from Figure 10 that the score distribution of each index analyzed by PLSR was relatively scattered, indicating that the samples with different processing treatments had a significant impact on the experimental results. The load diagram of PLSR analysis was shown in Figure 13, in which principal factor 1 explains 15% of the total variation, and principal factor 2 explains 14% of the total variation. Principal components 1 and 2 explain 36% and 13% of the Y variable, respectively. It can be seen that the fatty acid composition of red vinasse-blue round scad after different processing during storage was mainly reflected in the first principal component.

It was reported that the PUFA/SFA value was generally used to evaluate the nutritional value of meat in nutrition; the higher the value, the better the nutritional value. At the same time, the higher the SFA + MUFA value, the better the tenderness, juiciness, and flavor of meat products, and the SFA + MUFA value can be used to measure the flavor and other related quality indicators after food processing [22,47,48]. Therefore, according to the results of the first principal component analysis, we can assume that the sample closer to the right side of the load diagram had better nutritional value of fatty acids, while the sample closer to the left side had better tenderness and juiciness. Therefore, the fatty acid changes in the control group (C) and the tin foil baking group (F) had higher similarity and better nutritional composition, which was closely related to most of the changes of PUFA. However, the fatty acid changes in boiling group (B) and microwave group (M) had higher similarity, which may have better texture and flavor, and the samples of these two groups were more closely related to the changes of SFA. By comparison, the relative content of fatty acids in the boiling group had the most significant change during the storage period.

**Figure 14:**
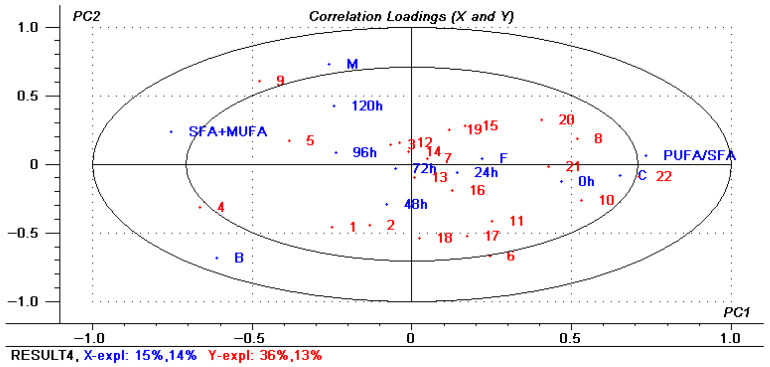
Loading plots of correlation of each index by PLSR analysis. The *X*-axis represented the main design variables: 12 of 0/1 sample variables (M: microwaving, B: boiling, F: foil baking, C: control group (non-processing), 6 time determination points, PUFA/SFA values, and SFA + MUFA values). The *Y*-axis represented the fatty acids of composition. Numbers 1 to 22 represented C18:2n-6 to C22:6n-3, respectively. The inner and outer circles represent correlation coefficients r^2^ = 0.5 (50%) and r^2^ = 1.0 (100%), respectively.

As can be seen from the analysis of Figure 14, with the extension of storage time, the relative content of PUFA in all groups showed a trend of overall decrease and overall increase of SFA from right to left. In terms of the changes of PUFA, 10(C18:2n-6), 20(C20:5n-3), 21(C22:6n-3), and 22(C22:6n-3) in the experimental group and control group were very close to PUFA/SFA in the first principal component, indicating that they all had a high relative content at the beginning of storage. With the prolongation of the storage period, it decreased significantly and was an important component affecting the nutritional composition of fatty acids of red vinasse-blue round scad. Similarly, in MUFA, 9 (C18:1n-9) and 5 (C16:1n-7) in the left half had an overall increasing trend with the extension of storage time; in particular, the change of C18:1n-9 was very significant, which was highly correlated with the regression model. Similarly, in the experimental group, the relative content of 1(C14:0), 2(C15:0), and 4(C16:0) in the left half of the loading diagram increased with the extension of storage time, while the relative content of 8 (C18:0) in the right half of the effect decreased with the extension of storage time. The above changes were consistent with the results of fatty acid composition changes (Table 7).

## 4. Conclusions

This study pioneered an intelligent optimization framework integrating Box–Behnken RSM with MATLAB analytics to advance functional foods development from red vinasse-blue round scad. The optimal parameters (the addition amount of red vinasse was 2.8 g/g, the processing temperature was 4 °C, and the processing time was 10 h) maximized product quality (good texture characteristics and sensory quality) through predictive control of biosynthesis technology-derived metabolites. The integrated optimization approach combining the Box–Behnken Design response surface methodology with MATLAB not only accurately determined the optimal extraction protocol but also visually identified reasonable processing parameter ranges. This provided both a theoretical foundation and innovative insights for red vinasse-blue round scad processing and industrial utilization research. Besides, the changes of lipids stability of the samples processed by microwaving, boiling, and foil baking during storage at 4 °C were studied. The total fat content of the baking group decreased the most and the MDA accumulation rate was the fastest during 4 °C refrigeration. In spite of this, the PUFA composition of red vinasse-blue round scad by different processing were all good and recommendable. The PLSR analysis showed that the relative content of fatty acids in the boiling group had the largest change, which was similar to that in microwave group. This research provided a transformative, intelligent optimization strategy for manufacturing shelf-stable, lipid-rich functional foods, bridging precision processing with nutraceutical preservation. Moreover, this work significantly enhanced the value of underutilized fish species, providing a scientific basis and a practical example for developing marine-based functional foods aimed at addressing specific health needs such as cardiovascular and cognitive health. It holds important practical significance for advancing the transformation toward a nutrition-oriented agricultural food system and improving public health. Meanwhile, the mechanism by which the addition of red vinasse affects the physicochemical properties of blue round scad remains to be further explored.

## Figures and Tables

**Table 1:** Scoring criteria for the sensory score.

Index	Scoring Standard	Scores
Appearance	The surface of the fillet looks clean, fresh, and non-shrinking.	16~20
	The surface cleanliness of the fillet was good, and the meat had a slight shrinkage phenomenon.	11~15
	The surface of the fillet had a sticky phenomenon, and the meat was slightly contracted and slimy.	6~10
	The surface of the fillet was seriously sticky, and the meat had obvious slippage.	0~5
Color	The fillets had a natural reddish color and were uniform.	26~30
	The color of the fillet was slightly red, and the color was more uniform.	20~25
	The fillets had uneven shades.	10~19
	The fillets were dark and uneven.	0~9
The aroma of red vinasse	The fillets had a slight wine aroma and a strong red vinasse aroma	12~15
	The fillets had a slight wine aroma and a general red vinasse aroma.	8~11
	The fillets had a slight wine aroma and a general red vinasse aroma.	4~7
	No obvious wine aroma and red vinasse aroma.	0~3
Unpleasant odor	No odor or fat oxidation.	12~15
	Light fishy smell or a small amount of fat oxidation.	8~11
	The fillets had a light fishy taste, and the fat oxidation taste was heavy.	4~7
	The fillets had a more pronounced fishy smell and fat oxidation smell.	0~3
Tissue	The fillets were firm, well textured, and elastic.	16~20
	The fillets were firmer, and the texture was clearer and more elastic.	11~15
	The fillets were not tight, locally looser, with poor elasticity.	6~10
	The fillets were soft and rotten, the loose phenomenon was heavier, and the fish had no elasticity.	0~5

**Table 2:** The factors and levels of the test.

Factors	Levels		
	−1	0	1
A: addition of red vinasse (g/g)	1:2.0	1:2.4	1:2.8
B: processing temperature (°C)	4	8	12
C: processing time (h)	10	12	14

**Table 3:** KMO and Bartlett sphere tests for dimensional reduction models of qualitative and structural indices.

KMO Measure of Sampling Adequacy		0.596
Bartlett’s Test of Sphericity	Approx. Chi-Square	15.179
	df	6
	Sig.	0.19

**Table 4:** The total variance of interpretation.

Component	Initial Eigenvalues			Extraction Sums of Squared Loadings		
	Total	% of Variance	Cumulative %	Total	% of Variance	Cumulative %
1	3.663	91.557	91.557	3.663	91.577	91.577
2	0.264	6.604	98.182			
3	0.069	1.723	99.905			
4	0.004	0.095	100.000			

Extraction method: principal component analysis.

**Table 5:** The design and result of response surface optimization.

No.	The Addition of Red Vinasse (g/g)	The Processing Temperature (°C)	The Processing Time (h)	Y1	Y2	Y
1	−1	−1	0	5.64	79.87	42.71
2	1	−1	0	5.82	81.79	43.69
3	−1	1	0	3.95	75.30	39.64
4	1	1	0	5.80	71.93	38.47
5	−1	0	−1	3.10	83.99	43.20
6	1	0	−1	5.94	85.84	45.72
7	−1	0	1	3.96	79.59	41.86
8	1	0	1	4.31	65.65	35.02
9	0	−1	−1	5.76	86.00	45.58
10	0	1	−1	3.82	72.20	38.41
11	0	−1	1	5.24	77.04	41.22
12	0	1	1	5.17	64.30	34.66
13	0	0	0	5.71	71.02	38.81
14	0	0	0	5.10	71.63	38.32
15	0	0	0	4.65	69.60	36.93
16	0	0	0	4.84	68.88	36.63
17	0	0	0	4.08	72.00	38.41

**Table 6:** Variance analysis of regression equation.

Sours	Sum of Squares	df	Mean Square	F Value	Prob > F	Significant
Model	174.2501	9	19.36112	11.75373	0.0019	**
A	2.548153	1	2.548153	1.54693	0.2536	
B	60.61005	1	60.61005	36.79509	0.0005	**
C	50.72763	1	50.72763	30.79568	0.0009	**
AB	1.161006	1	1.161006	0.704823	0.4289	
AC	21.85563	1	21.85563	13.26809	0.0083	**
BC	0.094556	1	0.094556	0.057403	0.8175	
A^2^	24.18195	1	24.18195	14.68035	0.0064	**
B^2^	3.507842	1	3.507842	2.129538	0.1879	
C^2^	6.411604	1	6.411604	3.892351	0.0891	
Residual	11.53062	7	1.647232			
Lack of Fit	7.748094	3	2.582698	2.731186	0.1781	Not
Pure Error	3.78253	4	0.945633			
Cor Total	185.7807	16				

Note: Prob > F value less than 0.01 indicated a significant difference and was marked as “**”.

**Table 7:** Effects of different processing methods on fatty acid composition of red vinasse-blue round scad.

Fatty Acids	Microwave						Boiling						Foil Baking						Control					
	0 h	24 h	48 h	72 h	96 h	120 h	0 h	24 h	48 h	72 h	96 h	120 h	0 h	24 h	48 h	72 h	96 h	120 h	0 h	24 h	48 h	72 h	96 h	120 h
C14:0	0.74 ± 0.01 b	0.61 ± 0.06 b	0.60 ± 0.13 bc	0.34 ± 0.04 b	0.46 ± 0.05 b	0.71 ± 0.04 b	0.74 ± 0.01 b	0.66 ± 0.15 ab	1.35 ± 0.05 a	1.16 ± 0.21 a	1.13 ± 0.17 a	0.79 ± 0.09 ab	0.63 ± 0.02 c	0.85 ± 0.06 ab	0.52 ± 0.04 c	0.70 ± 0.00 b	1.15 ± 0.14 a	0.76 ± 0.03 ab	0.65 ± 0.02 c	0.89 ± 0.00 a	0.79 ± 0.04 b	0.67 ± 0.15 b	0.72 ± 0.21 ab	0.90 ± 0.06 a
C15:0	0.25 ± 0.01 a	0.15 ± 0.00 c	0.14 ± 0.07 a	0.20 ± 0.04 b	0.12 ± 0.01 b	0.10 ± 0.05 b	0.25 ± 0.01 a	0.25 ± 0.00 a	0.25 ± 0.03 a	0.34 ± 0.00 a	0.31 ± 0.05 a	0.13 ± 0.02 b	0.20 ± 0.04 a	0.22 ± 0.04 ab	0.13 ± 0.04 a	0.18 ± 0.06 b	0.27 ± 0.05 a	0.41 ± 0.17 a	0.24 ± 0.02 a	0.18 ± 0.00 bc	0.22 ± 0.01 a	0.13 ± 0.08 b	0.15 ± 0.01 b	0.15 ± 0.01 b
C15:1	1.12 ± 0.09 ab	1.16 ± 0.03 a	1.33 ± 0.09 a	1.46 ± 0.03 a	1.08 ± 0.06 a	1.20 ± 0.00 b	1.12 ± 0.09 ab	1.21 ± 0.02 a	1.26 ± 0.08 a	1.34 ± 0.01 bc	1.02 ± 0.11 a	1.15 ± 0.05 b	1.26 ± 0.03 a	1.05 ± 0.06 b	1.34 ± 0.05 a	1.40 ± 0.03 ab	1.19 ± 0.03 a	2.54 ± 0.33 a	1.19 ± 0.03 ab	1.00 ± 0.02 b	1.30 ± 0.03 a	1.28 ± 0.06 c	1.01 ± 0.04 a	0.90 ± 0.00 b
C16:0	18.98 ± 0.68 a	19.09 ± 0.29 b	21.46 ± 0.30 c	20.74 ± 0.49 b	20.14 ± 0.02 b	23.87 ± 0.09 a	18.98 ± 0.68 a	22.58 ± 0.15 a	24.05 ± 0.10 a	25.06 ± 0.13 a	25.78 ± 1.01 a	23.12 ± 0.38 a	19.55 ± 0.41 a	19.17 ± 0.20 b	20.22 ± 0.02 d	18.76 ± 0.73 c	24.84 ± 1.33 a	14.65 ± 0.42 c	17.69 ± 0.08 b	19.65 ± 0.28 b	22.04 ± 0.25 b	20.71 ± 0.47 b	17.30 ± 0.41 c	19.67 ± 0.77 b
C16:1 n-7	1.35 ± 0.73 a	1.50 ± 0.00 ab	1.59 ± 0.21 bc	1.30 ± 0.11 ab	1.85 ± 0.10 ab	2.67 ± 0.07 b	1.35 ± 0.73 a	1.28 ± 0.00 b	2.21 ± 0.07 a	2.01 ± 0.01 a	3.35 ± 0.13 a	2.36 ± 0.24 bc	1.66 ± 0.31 a	1.39 ± 0.11 ab	1.80 ± 0.00 b	0.60 ± 0.55 b	2.07 ± 1.59 ab	6.19 ± 0.05 a	0.79 ± 0.01 a	1.78 ± 0.29 a	1.28 ± 0.13 c	1.42 ± 0.05 a	0.36 ± 0.01 b	2.15 ± 0.05 c
C17:0	1.41 ± 0.08 a	0.88 ± 0.00 d	1.09 ± 0.00 b	1.02 ± 0.14 b	0.85 ± 0.02 b	0.76 ± 0.04 a	1.41 ± 0.08 a	1.37 ± 0.01 a	1.12 ± 0.01 c	1.11 ± 0.03 b	1.06 ± 0.11 a	1.00 ± 0.13 a	1.28 ± 0.01 a	1.17 ± 0.00 b	1.04 ± 0.02 d	1.36 ± 0.01 a	1.04 ± 0.01 a	0.77 ± 0.15 a	1.29 ± 0.03 a	1.09 ± 0.01 c	1.18 ± 0.00 a	0.79 ± 0.04 c	0.99 ± 0.06 ab	0.95 ± 0.07 a
C17:1	0.61 ± 0.06 b	0.60 ± 0.06 b	0.82 ± 0.01 a	0.84 ± 0.03 a	0.58 ± 0.04 ab	0.60 ± 0.00 b	0.61 ± 0.06 b	0.78 ± 0.04 a	0.61 ± 0.01 b	0.79 ± 0.12 a	0.45 ± 0.02 c	0.60 ± 0.07 b	0.78 ± 0.03 a	0.64 ± 0.00 b	0.64 ± 0.04 b	0.59 ± 0.03 b	0.60 ± 0.02 a	1.07 ± 0.05 a	0.66 ± 0.01 b	0.60 ± 0.03 b	0.82 ± 0.00 a	0.67 ± 0.04 ab	0.51 ± 0.03 bc	0.45 ± 0.00 c
C18:0	14.27 ± 0.11 a	12.96 ± 0.10 b	12.76 ± 0.21 b	12.69 ± 0.05 a	14.26 ± 0.00 a	12.20 ± 0.05 b	14.27 ± 0.11 a	13.82 ± 0.11 a	10.79 ± 0.07 c	9.16 ± 0.67 b	10.38 ± 0.97 c	12.02 ± 0.03 b	13.11 ± 0.50 b	13.61 ± 0.03 a	13.14 ± 0.22 ab	12.86 ± 0.26 a	9.57 ± 0.69 c	11.91 ± 0.02 b	14.55 ± 0.24 a	13.10 ± 0.09 b	13.36 ± 0.04 a	12.28 ± 0.08 a	12.12 ± 0.14 b	13.17 ± 0.27 a
C18:1 n-9 c	8.35 ± 0.24 b	14.52 ± 0.31 a	10.25 ± 0.06 b	12.40 ± 0.00 b	14.80 ± 0.03 a	15.64 ± 0.09 a	8.35 ± 0.24 b	9.09 ± 0.05 c	12.35 ± 0.05 a	10.51 ± 0.05 c	13.74 ± 1.68 a	11.19 ± 1.07 b	8.34 ± 1.21 b	8.65 ± 0.27 c	10.37 ± 0.43 b	10.27 ± 1.14 c	13.30 ± 0.20 a	14.90 ± 0.12 a	7.30 ± 0.12 b	10.34 ± 0.06 b	8.78 ± 0.15 c	14.26 ± 0.08 a	10.06 ± 0.16 b	12.71 ± 0.36 b
C18:2 n-6 c	3.01 ± 0.82 ab	2.02 ± 0.04 a	2.57 ± 0.08 b	2.03 ± 0.06 b	1.97 ± 0.19 b	1.52 ± 0.09 b	3.01 ± 0.82 ab	2.74 ± 0.53 a	1.57 ± 0.03 d	1.92 ± 0.01 b	1.78 ± 0.11 b	1.81 ± 0.15 b	3.67 ± 0.09 a	2.68 ± 0.23 a	3.12 ± 0.04 a	2.62 ± 0.01 a	1.69 ± 0.24 b	1.68 ± 0.27 b	2.43 ± 0.17 ab	2.69 ± 0.04 a	2.40 ± 0.04 c	1.61 ± 0.10 c	3.38 ± 0.17 a	2.65 ± 0.04 a
C18:3 n-6	0.75 ± 0.03 b	0.37 ± 0.00 b	0.28 ± 0.15 a	0.27 ± 0.03 c	0.19 ± 0.06 b	0.33 ± 0.07 b	0.75 ± 0.03 b	0.29 ± 0.12 b	0.46 ± 0.08 a	0.72 ± 0.02 a	0.44 ± 0.06 a	0.31 ± 0.01 b	0.64 ± 0.00 c	0.59 ± 0.01 a	0.28 ± 0.13 a	0.40 ± 0.04 b	0.44 ± 0.03 a	0.47 ± 0.01 a	0.86 ± 0.03 a	0.40 ± 0.03 b	0.20 ± 0.00 a	0.30 ± 0.01 c	0.31 ± 0.12 ab	0.37 ± 0.04 ab
C18:3 n-3	0.32 ± 0.04 b	0.29 ± 0.02 b	0.24 ± 0.01 b	0.36 ± 0.04 b	0.43 ± 0.00 a	0.22 ± 0.01 a	0.32 ± 0.04 b	0.27 ± 0.01 c	0.38 ± 0.02 a	0.52 ± 0.07 a	0.30 ± 0.10 ab	0.27 ± 0.02 a	0.48 ± 0.01 b	0.45 ± 0.02 ab	0.27 ± 0.02 b	0.34 ± 0.02 bc	0.39 ± 0.06 a	0.50 ± 0.22 a	0.22 ± 0.03 b	0.23 ± 0.02 c	0.24 ± 0.00 b	0.24 ± 0.02 c	0.20 ± 0.01 b	0.40 ± 0.00 a
C20:1 n-9	0.12 ± 0.00 a	0.08 ± 0.02 a	0.07 ± 0.00 b	0.10 ± 0.02 a	0.06 ± 0.01 c	0.08 ± 0.02 a	0.12 ± 0.00 a	0.09 ± 0.03 a	0.16 ± 0.01 a	0.20 ± 0.07 a	0.13 ± 0.04 ab	0.07 ± 0.01 a	0.10 ± 0.00 ab	0.48 ± 0.57 a	0.08 ± 0.00 b	0.14 ± 0.07 a	0.18 ± 0.00 a	0.31 ± 0.22 a	0.10 ± 0.03 ab	0.09 ± 0.01 a	0.05 ± 0.00 c	0.07 ± 0.01 a	0.08 ± 0.00 bc	0.06 ± 0.00 a
C20:2 n-6	0.46 ± 0.03 b	0.20 ± 0.01 c	0.33 ± 0.03 a	0.70 ± 0.00 a	0.64 ± 0.00 a	0.21 ± 0.01 b	0.46 ± 0.03 b	0.29 ± 0.03 b	0.24 ± 0.01 b	0.50 ± 0.00 b	0.39 ± 0.16 ab	0.30 ± 0.05 b	0.34 ± 0.01 bc	0.40 ± 0.01 a	0.22 ± 0.00 b	0.36 ± 0.02 c	0.23 ± 0.08 b	0.55 ± 0.04 a	0.68 ± 0.12 a	0.25 ± 0.01 b	0.25 ± 0.03 b	0.21 ± 0.04 d	0.28 ± 0.02 b	0.27 ± 0.04 b
C22:0	0.13 ± 0.00 b	0.16 ± 0.01 a	0.12 ± 0.00 ab	0.11 ± 0.01 ab	0.13 ± 0.00 a	0.10 ± 0.02 b	0.13 ± 0.00 b	0.08 ± 0.01 c	0.10 ± 0.01 ab	0.09 ± 0.00 bc	0.13 ± 0.06 a	0.09 ± 0.02 b	0.13 ± 0.00 b	0.13 ± 0.00 b	0.13 ± 0.02 a	0.08 ± 0.00 c	0.10 ± 0.01 a	0.38 ± 0.11 a	0.16 ± 0.02 b	0.13 ± 0.01 b	0.09 ± 0.01 b	0.12 ± 0.01 a	0.12 ± 0.00 a	0.08 ± 0.00 b
C20:3 n-6	4.00 ± 0.15 b	4.39 ± 0.01 a	3.50 ± 0.02 c	3.68 ± 0.03 b	3.62 ± 0.00 b	3.54 ± 0.10 b	4.00 ± 0.15 b	3.97 ± 0.00 c	4.26 ± 0.03 a	4.02 ± 0.18 a	3.89 ± 0.10 a	3.57 ± 0.15 b	4.11 ± 0.20 b	4.28 ± 0.03 b	3.63 ± 0.18 c	4.02 ± 0.08 a	3.72 ± 0.05 ab	3.95 ± 0.09 a	3.80 ± 0.07 b	3.65 ± 0.04 d	3.96 ± 0.01 b	4.11 ± 0.06 a	3.19 ± 0.08 c	3.99 ± 0.09 a
C20:3 n-3	0.26 ± 0.02 a	0.24 ± 0.00 a	0.20 ± 0.01 b	0.15 ± 0.01 c	0.14 ± 0.02 b	0.14 ± 0.01 a	0.26 ± 0.02 a	0.23 ± 0.01 a	0.31 ± 0.01 a	0.33 ± 0.02 a	0.18 ± 0.03 ab	0.18 ± 0.06 a	0.23 ± 0.03 a	0.24 ± 0.00 a	0.22 ± 0.04 b	0.17 ± 0.04 bc	0.26 ± 0.06 a	0.26 ± 0.13 a	0.27 ± 0.04 a	0.25 ± 0.01 a	0.21 ± 0.01 b	0.23 ± 0.00 b	0.16 ± 0.03 b	0.22 ± 0.00 a
C20:4 n-6	6.07 ± 0.12 a	5.56 ± 0.03 c	6.59 ± 0.13 b	4.13 ± 0.20 d	3.77 ± 0.07 c	5.11 ± 0.04 a	6.07 ± 0.12 a	5.03 ± 0.03 d	7.00 ± 0.06 a	7.00 ± 0.04 a	6.67 ± 0.45 ab	5.68 ± 0.01 a	5.85 ± 0.02 ab	6.05 ± 0.08 b	7.14 ± 0.01 a	6.70 ± 0.04 b	6.89 ± 0.42 a	4.75 ± 0.95 a	5.78 ± 0.10 bc	6.68 ± 0.07 a	5.03 ± 0.00 c	5.42 ± 0.05 c	5.92 ± 0.03 b	5.96 ± 0.15 a
C22:2 n-6	0.45 ± 0.07 b	0.47 ± 0.19 a	0.27 ± 0.03 b	0.82 ± 0.08 a	0.58 ± 0.01 a	0.31 ± 0.01 b	0.45 ± 0.07 b	0.55 ± 0.04 a	0.44 ± 0.07 a	0.37 ± 0.00 b	0.37 ± 0.02 b	0.26 ± 0.02 b	0.51 ± 0.05 b	0.52 ± 0.07 a	0.31 ± 0.07 ab	0.41 ± 0.13 b	0.31 ± 0.05 bc	0.75 ± 0.17 a	0.48 ± 0.03 b	0.48 ± 0.00 a	0.39 ± 0.01 ab	0.38 ± 0.03 b	0.26 ± 0.04 c	0.32 ± 0.01 b
C20:5 n-3	2.41 ± 0.07 b	2.29 ± 0.07 a	1.91 ± 0.07 a	2.91 ± 0.02 a	2.73 ± 0.06 a	1.65 ± 0.08 b	2.41 ± 0.07 b	2.00 ± 0.11 b	1.61 ± 0.08 b	1.51 ± 0.01 c	1.42 ± 0.02 c	1.62 ± 0.11 b	2.12 ± 0.04 c	2.13 ± 0.08 ab	1.95 ± 0.01 a	1.89 ± 0.11 b	1.33 ± 0.02 c	2.54 ± 0.30 a	2.61 ± 0.08 a	2.11 ± 0.11 ab	1.99 ± 0.03 a	2.02 ± 0.02 b	2.34 ± 0.12 b	1.58 ± 0.09 b
C22:5 n-3	1.93 ± 0.08 c	2.12 ± 0.00 a	2.00 ± 0.10 ab	1.59 ± 0.09 c	1.64 ± 0.02 a	1.74 ± 0.12 a	1.93 ± 0.08 c	1.91 ± 0.01 d	1.90 ± 0.11 b	1.75 ± 0.01 bc	1.54 ± 0.11 a	2.08 ± 0.24 a	2.11 ± 0.02 b	2.06 ± 0.02 b	2.12 ± 0.03 a	2.38 ± 0.25 a	1.53 ± 0.01 a	1.84 ± 0.10 a	1.97 ± 0.02 c	2.01 ± 0.03 c	1.80 ± 0.01 b	2.04 ± 0.15 ab	1.78 ± 0.14 a	2.00 ± 0.21 a
C22:6 n-3	33.01 ± 1.18 b	30.34 ± 0.26 d	31.88 ± 0.20 b	32.16 ± 0.23 ab	29.97 ± 0.10 b	27.30 ± 0.22 b	33.01 ± 1.18 b	31.50 ± 0.08 c	27.57 ± 0.13 d	29.55 ± 0.15 b	25.54 ± 0.56 c	31.41 ± 0.76 a	32.90 ± 0.99 b	33.22 ± 0.12 a	31.32 ± 0.17 c	33.78 ± 2.16 a	28.89 ± 1.72 b	28.81 ± 0.26 b	36.29 ± 0.28 a	32.41 ± 0.28 b	33.59 ± 0.07 a	31.04 ± 1.14 ab	38.76 ± 0.18 a	31.06 ± 1.25 a
SFA	35.79 ± 0.89 a	33.84 ± 0.46 c	36.17 ± 0.72 b	35.09 ± 0.78 b	35.95 ± 0.10 b	37.74 ± 0.30 a	35.79 ± 0.89 a	38.77 ± 0.44 a	37.67 ± 0.27 a	36.93 ± 1.04 a	38.78 ± 2.37 a	37.14 ± 0.66 a	34.91 ± 0.99 b	35.15 ± 0.33 b	35.18 ± 0.35 c	33.94 ± 1.06 b	36.95 ± 2.23 b	28.88 ± 0.89 c	34.57 ± 0.41 bc	35.04 ± 0.40 b	37.69 ± 0.35 a	34.70 ± 0.83 b	31.39 ± 0.83 c	34.92 ± 1.18 b
MUFA	11.55 ± 1.13 b	17.86 ± 0.43 a	14.06 ± 0.37 b	16.10 ± 0.19 ab	18.38 ± 0.23 a	20.18 ± 0.19 b	11.55 ± 1.13 b	12.45 ± 0.14 c	16.59 ± 0.22 a	14.86 ± 0.27 bc	18.70 ± 1.97 a	15.38 ± 1.44 c	12.14 ± 1.59 b	12.22 ± 1.01 c	14.23 ± 0.52 b	12.99 ± 1.82 c	17.34 ± 1.84 a	25.01 ± 0.77 a	10.04 ± 0.19 c	13.81 ± 0.41 b	12.24 ± 0.31 c	17.70 ± 0.24 a	12.03 ± 0.24 b	16.26 ± 0.42 c
PUFA	52.66 ± 2.60 b	48.29 ± 0.65 c	49.77 ± 0.84 a	48.81 ± 0.79 b	45.67 ± 0.53 b	42.08 ± 0.75 c	52.66 ± 2.60 b	48.79 ± 0.97 c	45.75 ± 0.63 b	48.20 ± 0.51 b	42.52 ± 1.72 b	47.48 ± 1.57 ab	52.95 ± 1.46 b	52.63 ± 0.65 a	50.58 ± 0.71 a	53.06 ± 2.88 a	45.71 ± 2.74 b	46.11 ± 2.53 b	55.39 ± 0.97 a	51.15 ± 0.63 b	50.07 ± 0.22 a	47.59 ± 1.64 b	56.58 ± 0.93 a	48.82 ± 1.93 a
n-6 PUFA	14.74 ± 1.22 a	13.01 ± 0.29 b	13.54 ± 0.45 b	11.64 ± 0.40 b	10.76 ± 0.34 b	11.03 ± 0.31 b	14.74 ± 1.22 a	12.87 ± 0.75 b	13.98 ± 0.29 b	14.54 ± 0.25 a	13.54 ± 0.90 a	11.92 ± 0.38 ab	15.11 ± 0.37 a	14.52 ± 0.42 a	14.70 ± 0.43 a	14.51 ± 0.32 a	13.29 ± 0.87 a	12.16 ± 1.53 ab	14.03 ± 0.53 ab	14.15 ± 0.19 a	12.23 ± 0.10 c	12.03 ± 0.29 b	13.34 ± 0.46 a	13.56 ± 0.37 a
n-3 PUFA	37.92 ± 1.38 b	35.28 ± 0.36 d	36.23 ± 0.39 b	37.17 ± 0.39 ab	34.91 ± 0.20 b	31.06 ± 0.43 b	37.92 ± 1.38 b	35.91 ± 0.22 c	31.77 ± 0.34 c	33.66 ± 0.26 b	28.98 ± 0.82 d	35.56 ± 1.19 a	37.84 ± 1.08 b	38.11 ± 0.23 a	35.88 ± 0.28 b	38.56 ± 2.57 a	32.41 ± 1.87 c	33.95 ± 1.01 a	41.37 ± 0.44 a	37.01 ± 0.44 b	37.84 ± 0.12 a	35.56 ± 1.34 ab	43.24 ± 0.48 a	35.26 ± 1.56 a
PUFA/SFA	1.47 ± 2.91 b	1.43 ± 1.41 b	1.38 ± 1.17 b	1.39 ± 1.01 b	1.27 ± 5.12 b	1.12 ± 2.48 c	1.47 ± 2.91 b	1.26 ± 2.19 c	1.21 ± 2.32 d	1.31 ± 0.49 b	1.10 ± 0.72 c	1.28 ± 2.37 b	1.52 ± 1.47 ab	1.50 ± 1.96 a	1.44 ± 2.04 a	1.56 ± 2.71 a	1.24 ± 1.23 b	1.60 ± 2.84 a	1.60 ± 2.34 a	1.46 ± 1.60 ab	1.33 ± 0.63 c	1.37 ± 1.96 b	1.80 ± 1.13 a	1.40 ± 1.64 b

Note: Different letters in the same row represent significant differences between the results (*p* < 0.05). PUFA: Polyunsaturated fatty acid; SFA: Saturated fatty acid; MUFA: Monounsaturated fatty acid.

## Data Availability

The original contributions presented in the study are included in the article; further inquiries can be directed to the corresponding author.
